# Disseminated intravascular coagulation initial score as a predictor of mortality in children with dengue shock syndrome: A retrospective cohort study

**DOI:** 10.1016/j.amsu.2022.103890

**Published:** 2022-06-10

**Authors:** Nurnaningsih, Samlek Elisawyn Sunbanu, Desy Rusmawatiningtyas, Eggi Arguni, Firdian Makrufardi, Intan Fatah Kumara

**Affiliations:** Department of Child Health, Faculty of Medicine, Public Health and Nursing, Universitas Gadjah Mada/Dr. Sardjito Hospital, Yogyakarta, 55281, Indonesia

**Keywords:** Children, Dengue shock syndrome, DIC initial Score, Mortality

## Abstract

**Background:**

The mortality of dengue shock syndrome (DSS) in children is still high at 12–44%. Assessment of DIC initial score using the International Society on Thrombosis and Haemostasis scoring system can help diagnosing and treating DIC, while also predicting mortality in pediatric patients with DSS.

**Methods:**

We retrospectively collected data of children with DSS at Dr. Sardjito Hospital between January 2017 and June 2021 with inclusion criteria such as age 1 month to 18 years and laboratory parameters taken within first 24 h after DSS workup.

**Results:**

Results showed a sample population consisting of 20 male subjects (58.8%), 24 aged >5 years (76.0%), 21 with good nutritional status (61.8%), and median length of stay 5 days (3–7), with saturation of 98% (97–99) and median pediatric Glasgow coma scale level of consciousness of 13. The laboratory profile showed median levels of hematocrit at 40.9% (32.9–44.9), thrombocytes at 20,500/L (14,000–32,000), prothrombin time of 17.8 s (14.9–25.3), fibrinogen at 123 mg/dL (106–184) and D-dimer at 832.5 ng/mL (362–1119). A DIC initial score of ≥5 25 (73.5%) resulted with a mortality of 9 children (36.0%) with a 92% survival rate in the first 6 h. The first 6-h survival according to each DIC score parameter showed 93.8%, 100%, 85.7%, and 94.1% of thrombocytes ≤50,000 μ/L, fibrinogen <100 mg/dL, D-dimer >1000 ng/mL, and prothrombin time >6 s, respectively.

**Conclusion:**

A DIC initial score ≥5 can be used as a mortality predictor in the first 6 h after DSS diagnosis.

## Highlights

• The mortality of dengue shock syndrome (DSS) in children is still high.
• Assessment of DIC initial score using the International Society on Thrombosis and Haemostasis scoring system.
• DIC initial score can be used as a mortality predictor.

## 1 Introduction

Dengue shock syndrome (DSS) is one of the dengue hemorrhagic fever (DHF) clinical manifestations which can increase patient morbidity and mortality through a series of immunological processes that result in plasma leakage and massive bleeding [1,2]. This viral infectious disease has a mortality rate of less than 1%, but if DSS occurs it can increase the mortality rate to 12–44% [3].

The underlying pathophysiology of DSS is an increase in vascular permeability resulting in large amounts of plasma leakage. The process of plasma leakage causes the volume of plasma in the blood vessels to decrease (hypovolemia) resulting in hemoconcentration which results in decreased blood flow in the peripheral vascular system. As a result, it has a negative impact on various important organs, from the central nervous system to the urinary system [3,4].

The disease known as DSS is a sum of symptoms and signs of shock that include tachycardia, hypotension, decreased thrombocyte count, fluid accumulation with respiratory distress, massive bleeding, organ failure or manifesting as cardiorespiratory system failure until cardiac arrest occurs [[5], [6]]. Massive bleeding is one of the most common complications in patients with DSS. The pathophysiology of massive bleeding in DSS includes the presence of severe thrombocytopenia, coagulation pathway dysfunction due to dengue virus infection in hepatocytes and D-dimer increment due to intravascular fibrinolysis from shock [6].

Disseminated intravascular coagulation (DIC) is one of the mortality predictors in DSS patients with acute kidney injury (AKI) which increases the risk by 2.483 times. The proportion of patients with DSS who died due to DIC was 82% [7,8]. The clinical manifestations of DIC in patients with DSS are related to thrombocyte counts, prolongation of coagulation factors during shock such as prolongation of prothrombin time (PT) and a D-dimer increment due to fibrinolysis, resulting in fibrinogen levels decrement [8,9].

The DIC initial score is a score applied on all patients admitted to the hospital with an initial diagnosis of DSS while in the emergency room or patients with dengue hemorrhagic fever (DHF) who are under treatment for DSS regardless whether the patient is diagnosed with DIC or not. The DIC scoring is based on the International Society on Thrombosis and Haemostasis (ISTH) scoring system, consisting of the number of thrombocytes, D-dimer, fibrinogen and PT taken at hospital admission up to <24 h of treatment with an initial diagnosis of DSS or DSS diagnosed during treatment and DIC parameters taken within 24 h since diagnosed with DSS [10].

Assessment of DIC initial score at the time diagnosed as DSS will also help establishing the diagnosis of DIC. The total score <5 is suggestive DIC and 5 is confirmed DIC. Early diagnosis of DIC when diagnosed with DSS is expected to help clinicians in managing shock in patients so that they can produce a good prognosis in the process of reducing morbidity and mortality rates [10]. This study was aimed to predict mortality of pediatric with DSS using DIC initial score.

## 2 Methods

### 2.1 Study design and population

This analytic observational study was conducted with a retrospective cohort design using secondary data on pediatric patients who have been diagnosed with DSS at Dr. Sardjito General Hospital, Yogyakarta from January 2017 to June 2021. Patients who were admitted to the PICU with DSS as one of their diagnosis were included in this study. The analysis excluded patients with missing medical records or inadequate data related to diagnosis criteria and evaluated factors. The sample selection method in this study used nonprobability sampling, namely consecutive sampling of all subjects whose DIC scores were calculated at the time of initial hospital admission with a diagnosis of DSS or diagnosed with DSS during treatment and laboratory parameters of DIC were taken in less than 24 h since diagnosis.

The variables in this study consisted of independent variables and dependent variables. The independent variable is the initial DIC score in the form of laboratory parameters consisting of thrombocytes, PT, D-dimer, and fibrinogen while the dependent variable is mortality. Data analysis was performed using SPSS version 22 computer program (IBM Corp., Armonk, NY). Demographic, clinical and laboratory data were analyzed using descriptive statistics which included frequency, percentage, mean and median. Bivariate analysis using Fisher's exact test was conducted to analyze the relationship between independent variables and mortality outcomes. Survival analysis using the Kaplan-Meier method was done to see the proportion of patients who were still alive after 6 h.

### Ethics approval

The research has been approved by the Medical and Health Research Ethics Committee of the Faculty of Medicine, Public Health and Nursing, Universitas Gadjah Mada, Yogyakarta, Indonesia with the number KE/FK/0603/EC/2021. This study was reported in line with the Strengthening the Reporting of Cohort Studies in Surgery (STROCSS) criteria [15].

## 3 Results

There were 158 children diagnosed with DSS from January 2017 to June 2021. A total of 34 children had laboratory data for DIC initial score calculation and met the eligibility criteria for the analysis of each independent variable.

Further analysis was done on 34 children who had complete data for DIC initial score calculation. The data on the characteristics of the research subjects can be seen in Table 1.

**Table 1:** Basic characteristics of research subjects.

Characteristic	Total (34)	
Age, n (%)		
<1 year old	4 (11.8)	
1–5 years old	6 (16.0)	
>5 years old	24 (76.0)	
Male, n (%)		20 (58.8)
Nutritional status, n (%)		
Poor		5 (14.7)
Good		21 (61.8)
Excellent		8 (23.5)
Referral, n (%)		33 (97)
Fever, median days (IQR)		5 (4-8)
Patient clinical parameter in ER		
Reactive pupils, n (%)		34 (100)
Ascites, n (%)		32 (94.1)
Hepatomegaly, n (%)		34 (100)
Gastrointestinal bleeding, n (%)		20 (58.8)
Cold extremities, n (%)		21 (61.8)
Assistive device usage, n (%)		6 (17.6)
Oxygenation, n (%)		
Non-rebreathing mask		1 (2.9)
Nasal cannula		24 (70.6)
Without oxygen supplementation		3 (8.8)
CRT >2 s, n (%)		19 (55.9)
SaO_2_ (%), median (IQR)		98 (97-99)
Consciousness level, median (IQR)		13 (11-15)
PELOD 2 score, median (IQR)		2 (2-11)
**Initial laboratory parameters within 24 h after DSS diagnosis**		
Hb gr/dL, median (IQR)	14.3 (11.5–15.3)	
Hct %, median (IQR)	40.9 (32.9–44.9)	
Leukocyte μ/L, median (IQR)	6720 (2080–35,740)	
Neutrophil %, mean	48.7 ± 10.8	
Lymphocyte %, mean	40.3 ± 13.2	
Monocyte %, median (IQR)	8.3 (5.4–11.2)	
NLR, median (IQR)	1.3 (0.8–1.7)	
Thrombocyte μ/L, median (IQR)	20,500 (14,000–32,000)	
PT seconds, median (IQR)	17.8 (14.9–25.3)	
Fibrinogen mg/dL, median (IQR)	123 (106-184)	
D-dimer ng/mL, median (IQR)	832.5 (362–1119)	
DIC initial score, median (IQR)	6 (4-7)	
*Length of stay,* median days (IQR)	5 (3-7)	
Mortality outcome, n (%)	9 (36.0)	

CRT, capillary refill time; ER, emergency room; DIC, disseminated intravascular coagulation;; PELOD, pediatric logistic organ dysfunction; SaO2, arterial oxygen saturation.

The number of male subjects was 20 (58.8%). The proportion of age >5 years was 24 (76%), good nutritional status 21 (61.8%), and emergency room admission with referral 33 (97%). The median days of fever during hospital admission were 5 days (4–8) in which 6 (17.6%) used an invasive breathing device. Twenty (58.8%) had gastrointestinal bleeding, 32 (94.1%) had ascites and 34 (100%) had hepatomegaly. Cold extremities were found in 21 (61.8%) with capillary refill time >2 s in 19 (55.9%). The initial laboratory profile of routine blood showed a mean neutrophil count of 48.7 ± 10.8%. The median thrombocyte count was 20,500/L (14,000–32,000). Median PT was 17.8 s (14.9–25.3). The median PELOD-2 score was 2 (2–11). The median value used in this analysis was caused by an abnormal distribution of the data.

There were 25 (73.5%) of the 34 research subjects who had DIC initial score of 5 based on the ISTH scoring system with a median DIC initial score of 6 (4–7). The median of each DIC component included thrombocytes 20,500/L (14,000–32,000), PT 17.8 s (14.9–25.3), fibrinogen 123 mg/dL (3–481), D-dimer 832.5 ng/mL (362–1119) with a median length of stay of 5 days (3–7) and total patient mortality of 9 (36.0%).

### 3.1 Bivariate analysis of the independent variables

Bivariate analysis was conducted on the DIC initial score and each component of the DIC score in Table 2 obtained from 34 research subjects. Each cell is added by 0.5 because there were groups that did not experience the effect so that the relative risk ratio (RR) value can be obtained.

The results of bivariate analysis showed that the effect of DIC initial score and each component of the DIC score on the mortality outcome were not statistically significant with an RR of 4.07 (95% CI: 0.59–28.14) and *p* > 0.05 which can be seen in Table 2.

**Table 2:** Bivariate analysis of DIC scores and components of DIC toward mortality.

Parameter	Death (n = 9)	Lived (n = 25)	RR	95% CI	*p*-value
DIC score					
≥5	9	16	4.07	0.59–28.14	0.124
<5	0	9			
Thrombocyte, μ/L					
≤50,000	9	23	1.18	0.19–6.95	1.00
51,000–100,000	0	2			
PT, seconds					
≥6	9	25			
<6	0	0			
D-dimer, ng/mL			2.86	0.86–9.54	0.116
>1000	6	8			
≤1000	3	17			
Fibrinogen, mg/mL					
≥100	7	19	1.08	0.28–4.18	1.00

CI, confidence interval; DIC, disseminated intravascular coagulation; PT; prothrombin time; RR, relative risk ratio.

### 3.2 Survival analysis based on DIC initial score and DIC component toward outcome

**Fig. 1:**
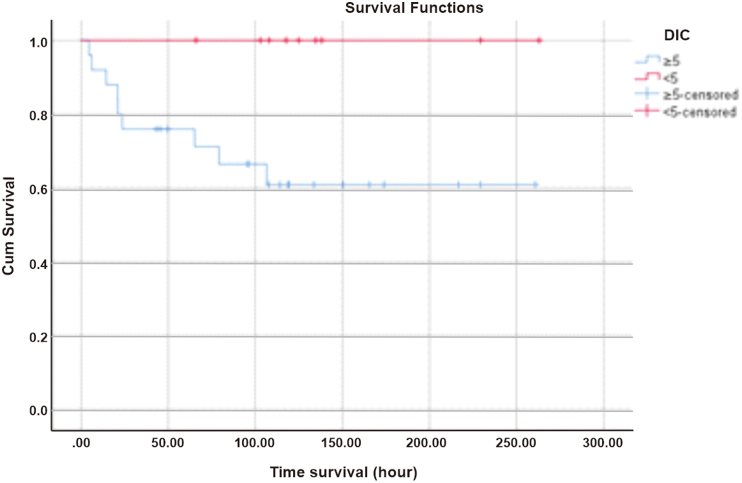
Survival based on DIC initial score.

The survival rate of pediatric patients with DIC initial score of 5 in the first 6 h since diagnosed with DSS at Dr. Sardjito General Hospital was 92%, persisted for 12 h, then decreased to 76% within 24 h of treatment as shown in Fig. 1.

**Fig. 2:**
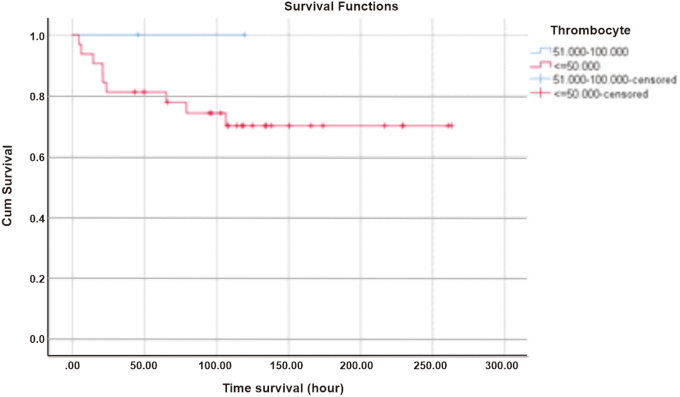
Survival based on thrombocyte count.

**Fig. 3:**
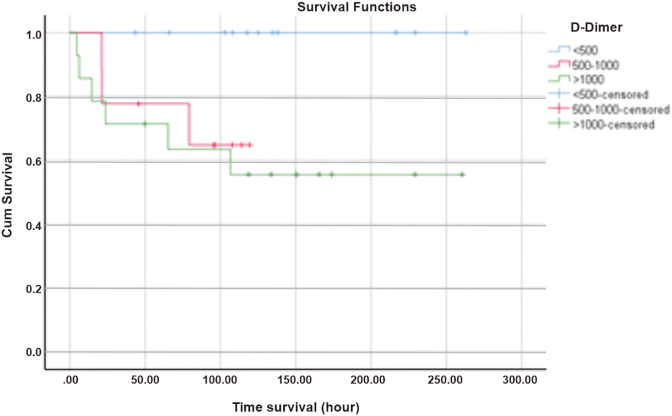
Survival based on D-Dimer level.

The 6-h survival in the thrombocyte 50,000/L group was 93.8% and remained until the 12th hour with a log rank of 0.458, then decreased to 81.3% at the 24th hour which can be seen in Fig. 2. D-dimer 500–1000 ng/mL survival at 6 h was 100% and decreased to 77.8% in the first 24 h, while D-dimer levels >1000 ng/mL at first 6 h had a survival rate of 85.7% with a log rank of 0.063 and became 71.4% at the 24th hour as shown in Fig. 3.

**Fig. 4:**
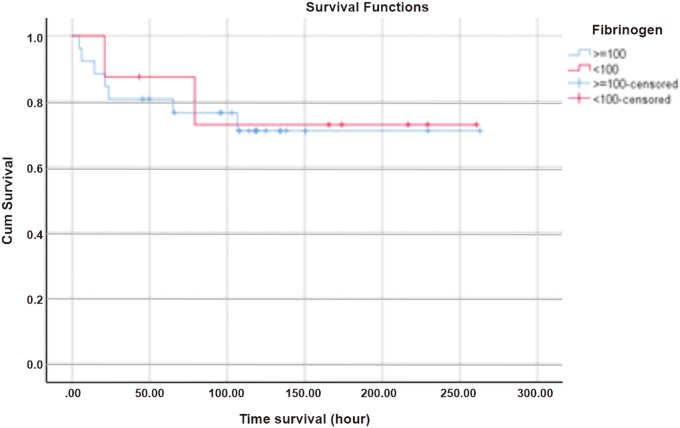
Survival based on fibrinogen level.

**Fig. 5:**
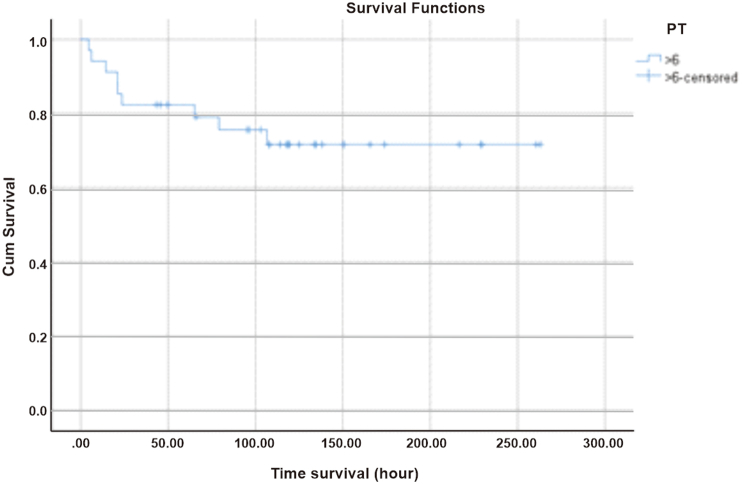
Survival based on prothrombin time.

Patients with fibrinogen levels <100 mg/dL at first 6 h had 100% survival with a log rank of 0.862 then decreased to 87.5% at 24th hours. Meanwhile, patients with fibrinogen levels 100 mg/dL had 92.3% survival at the 6th hour and remained constant until the 12th hour with a mean of 198.156 h (95% CI: 157.106–239.2) which had a log rank of 0.862, then decreased to 80.8% at the 24th hour as shown in Fig. 4. Subjects with a PT > 6 s had a survival rate of 94.1% at the 6th hour and remained until the 12th hour with a mean of 200.201 h (95% CI: 165.016–235.386), then decreased at the 4th hour by 82.4%, as shown in Fig. 5.

## 4 Discussion

Dengue virus infection is still a global problem. Dengue shock syndrome (DSS) is a complication of dengue virus infection that can increase mortality and morbidity. In this study, we found that the proportion of patients who died from DSS was 36%. The results obtained are in accordance with the research of Mishra et al. including 97 cases in India stated that the mortality rate due to dengue virus infection is generally less than 1% but if shock occurs it will increase the risk of mortality to 12%–44% [3].

Disseminated intravascular coagulation (DIC) is a predictor factor that increases mortality if shock has occurred in patients with DSS. Based on research conducted by Sari et al., in 2018 using retrospective study design at Dr. Sardjito General Hospital, it was found that in 65 patients DIC will increase the risk of death by 2.48 times. Their results were in line with this study in which 9 (36%) of 34 pediatric DSS patients with DIC initial score of 5 based on the International Society on Thrombosis and Haemostasis (ISTH) died. In the results of the bivariate analysis, there was no significant relationship between the DIC initial score of 5 and the initial score <5 on the death outcome with an RR of 4.07 (95% CI: 0.59–28.14, *p* = 0.124). The results of statistical analysis are not meaningful because the number of samples is not sufficient so that the power to find significant differences in the population is reduced but in fact the DIC initial score of 5 will increase the death risk higher than the score <5 [7].

The results of this study are also in line with the research conducted by Pangaribuan et al., in 2018 in the same hospital on 32 patients with DSS who experienced DIC and 27 (82%) of them died. From the results, when correlated with this study, DIC is one of the predictors of mortality in DSS. The results of survival analysis in 6 h and 12 h since DSS was diagnosed at Dr. Sardjito Hospital in patients with thrombocyte counts 50,000/L and PT > 6 s showed that patients had survival rates of 93.8% and 94.1%, respectively. Meanwhile, patients with D-dimer levels >1000 ng/mL in the first 6 h had a much lower survival, which was around 85.7%. This explains that if there is a high level of D-dimer in the blood, there has been severe intravascular coagulation that can accelerate multiorgan failure due to poor perfusion [6].

Research conducted by Khemani et al., in 2009 on 132 pediatric patients with shock and sepsis showed that a DIC score of 5 had a 50% mortality risk compared to a score <5 which had a 20% mortality risk. It was also found in this study, that 36% of pediatric patients with DIC who had a DIC score of 5 died compared to no deaths (0) in pediatric patients with a DIC score <5. This situation explains that the DIC initial score at the time of diagnosis of shock will be very helpful in the management and prognosis of DSS patients. If clinically there are signs of multiorgan failure, the DIC process that occurs in the patient is caused by the hypercoagulation process, not by the hyperfibrinolysis process. However, if the hyperfibrinolysis process characterized by blood fibrinogen levels <100 mg/dL occurs concurrently with hypercoagulation, the observed clinical manifestation is massive bleeding. This is in accordance with this study, in which 20 (58.8%) patients experienced massive bleeding in the gastrointestinal tract [11].

There are several laboratory parameters for calculating DIC scores to establish the diagnosis of DIC in addition to the presence of supporting clinical evidence. These parameters are thrombocyte count, PT, D-dimer levels, and fibrinogen levels based on the ISTH scoring system. The results of the bivariate analysis of each component of the DIC score showed no statistically significant difference. After *post hoc* power analysis, it was found that the amount of power in this study was 71.8%.

The recent results of this analysis are different from the research by Baiduri et al. including 67 pediatric patients in Surabaya with the results that there was a relationship between the severity of dengue infection in the form of disturbances in PT and activated partial thromboplastine time (aPTT) which had an RR of 2.27 times and 6.9 times (95% CI: 1.4–3.7 and 2.3–20.6). This study only observed one group, namely the DSS group and could not proceed to multivariate analysis because there was no significant difference between the two groups in bivariate analysis. No statistical significance occurred because all research subjects showed PT prolongation >6 s so it was not possible to know the PT value that played a role as predictor factor in mortality. However, PT played a role in patient survival in the first 6 h, which was 94.1% of patients were still alive and decreased to 82.4% at 24 h. This shows that PT prolongation is closely related to the presence of an unresolved shock process [12].

Thrombocytes also play a role in bleeding. Bleeding that occurs in patients with DSS can be through several pathways such as vascular resistance and coagulation factors. If there is a bleeding process with poor vascular resistance, it will result in prolonged shock so that it interferes with tissue perfusion. When shock occurs due to plasma leakage, perfusion in certain organs will decrease. Decreased perfusion due to poor vascular resistance and low thrombocyte count will result in bleeding that triggers severe hypovolemia. Severe hypovolemia can cause perfusion in organs to worsen and result in death in a fairly rapid time [13].

Thrombocyte count is a laboratory parameter that is often found in children with dengue infection without or accompanied by severe clinical manifestations. Low thrombocyte count is associated with suppression of the bone marrow due to dengue virus infection. A decrease in the number of leukocytes and an increase in lymphocytes were also observed in patients with dengue infection. The results of this study showed that there was no significant statistical relationship between thrombocyte count and mortality (95% CI: 0.19–6.95, *p* = 1.00). These results are in line with research conducted by Baiduri et al. that found the thrombocyte count is not a prognostic factor with an RR of 0.46 (95% CI: 0.001–291). These results are based on an analysis of the severe dengue and non-severe dengue groups, while in this study analysis was conducted only on the DSS group which was a type of severe dengue. A rapid decrease in thrombocyte count is associated with the severity of dengue virus infection as observed in this study, in which 93.8% of patients with a thrombocyte count 50,000/L in the first 6 h after DSS diagnosis were still alive but decreased to 81.3% after 24 h of treatment. This is closely related to bleeding manifestations, such as gastrointestinal bleeding that occurs in 58.8% of patients [12].

D-dimer is the end product of the fibrinolysis process that occurs due to various processes, one of which is shock. The D-dimer in the DIC score plays a role in determining the number of fibrinolytic process occurrences caused by intravascular coagulation. The level of D-dimer in plasma is directly proportional to the degree of shock and the body's ability to respond to shock. The research of Kheimani et al. is in line with this study which showed that the survival of DSS patients with D-dimer >1000 ng/mL at 6 h was 85.7% of which 14.3% of patients had died during that time. D-dimer is the component of the DIC score that contributes the most to patient survival in the first 6 h. The results of the analysis showed that almost all patients in this study had DIC which was characterized by high levels of D-dimer in serum [11].

Fibrinogen (Factor I) is a beta globin protein synthesized by the liver as an acute phase reactant that is useful for stimulating thrombus formation in the event of bleeding. Decreased fibrinogen levels indicate a high intravascular fibrinolysis process due to coagulation. The survival of DSS patients with fibrinogen levels <100 mg/dL at 6 h is 100% with a log rank of 0.862 and statistically not significant, but clinically a decrease in the amount of fibrinogen in plasma will result in massive bleeding due to a combination of hyperfibrinolysis and organ failure caused by hypercoagulation [13].

PT prolongation in DSS is associated with injury in hepatocytes due to dengue virus infection. Research conducted by Bashir et al. stated that PT and aPTT were predictors of coagulation disorders in patients with dengue infection at the beginning of the disease course before the onset of shock. The results of the bivariate analysis showed that there was no significant relationship between PT and death because patients who lived or died had PT > 6 s. The results of this study can only describe the survival of patients with PT > 6 s in 6 h, which is 94.1% [14].

The results of the bivariate analysis of the DIC initial score and the components of the DIC score based on ISTH were not statistically significant to the mortality outcome. The results of the survival analysis showed that patients with D-dimer components >1000 ng/mL (85.7%) and fibrinogen <100 mg/dL (100%) were still alive 6 h after DSS was diagnosed at Dr. Sardjito General Hospital. The thrombocyte and PT components suggest that approximately 6% of patients will die within 6 h. If all components of the DIC initial score were analyzed for survival, the patient's 6-h survival was approximately 92%, which means 8% of patients would die. This number will double at 24th hours if the shock is not resolved and the intravascular coagulation process is still ongoing.

This study used secondary data so that it has limitations and biases in measurement. The measurement bias encountered is in the clinical data of the patient, while the laboratory data uses examination tools that have been routinely calibrated from the clinical laboratory at Dr. Sardjito General Hospital, Yogyakarta, Indonesia. Another weakness of this research is the limited number of research samples so that the analysis results are obtained with a wide confidence range. This study is expected to provide a general description that coagulation factors and DIC are predictors of mortality in pediatric patients with DSS.

### 4.1 Future directions

This study gives potential pathophysiological mechanism behinds DSS pediatric patients. We recommend physician who treated such patients should notice DIC initial score to predict mortality. A further retrospective multicenter study that includes other confounders is essential to provide more realistic survival data on PICU patients with dengue shock syndrome which would help for appropriate risk stratification and therapeutic strategies [16]. Furthermore, treatment approaches may be further tailored to prevent progression of the disease.

## 5 Conclusions

A DIC initial score of 5 at the time diagnosed with DSS was a predictor of mortality compared to DIC initial score of <5 without death. In addition, the D-dimer level >1000 ng/mL is a component of the DIC score which has lower survival rate compared to the other three components of the DIC score, namely thrombocyte count, PT, and fibrinogen levels.

A cross-sectional diagnostic study can be conducted to determine the cut-off point of the DIC initial score in patients with dengue infection with clinical manifestations of DF, DHF and DSS. Thus, it can be clearly seen what DIC initial score for each clinical manifestation. In addition, it can determine which components of the DIC score (thrombocytes, PT, D-dimer, and fibrinogen) will affect each clinical manifestation of dengue virus infection.

## Ethical Approval

This study has been approved by the Ethical Committee of Faculty of Medicine, Public Health and Nursing, Universitas Gadjah Mada/Dr. Sardjito Hospital (Ref: KE/FK/0603/EC/2021)

## Funding

This research did not receive any specific grant from funding agencies in the public, commercial, or not-for-profit sectors.

## Consent

Written informed consent was obtained from the parents before joining the study. A copy of the written consent is available for review by the Editor-in-Chief of this journal on request.

## Author contribution

Nurnaningsih, Samlek Elisawyn Sunbanu, Desy Rusmawatingintyas, Eggi Arguni, and Intan Fatah Kumara conceived the study and critically revised the manuscript for important intellectual content. Nurnaningsih, Samlek Elisawyn Sunbanu, Desy Rusmawatingintyas, Eggi Arguni, and Firdian Makrufardi drafted the manuscript and critically revised the manuscript for important intellectual content. All authors read and approved the final draft. All authors facilitated all project-related tasks.

## Registration of Research Studies

researchregistry7950

## Provenance and peer review

Not commissioned, externally peer reviewed.

## Guarantor

Nurnaningsih.

## Declaration of competing interest

No potential conflict of interest relevant to this article was reported.
